# First Record of Mosquito-Borne Kyzylagach Virus in Central Europe

**DOI:** 10.3390/v12121445

**Published:** 2020-12-16

**Authors:** Silvie Šikutová, Patrik Dočkal, Petra Straková, Jan Mendel, Oldřich Šebesta, Lenka Betášová, Hana Blažejová, Zdeněk Hubálek, Ivo Rudolf

**Affiliations:** 1 Institute of Vertebrate Biology, The Czech Academy of Sciences, v.v.i., Květná 8, CZ-60365 Brno, Czech Republic; sikutova@ivb.cz (S.Š.); dockal@muf-pro.cz (P.D.); strakova.p@centrum.cz (P.S.); JMendel@seznam.cz (J.M.); oldrich.sebesta@khsbrno.cz (O.Š.); betasova@ivb.cz (L.B.); Hana.Blazejova@vetmeduni.ac.at (H.B.); zhubalek@brno.cas.cz (Z.H.); 2 Veterinary Research Institute, Hudcova 70, CZ-62100 Brno, Czech Republic

**Keywords:** alphaviruses, Sindbis, mosquito, *Culex modestus*, arboviruses, reedbeds

## Abstract

RNA of Kyzylagach virus (KYZV), a Sindbis-like mosquito-borne alphavirus from Western equine encephalitis virus complex, was detected in four pools (out of 221 pools examined), encompassing 10,784 female *Culex modestus* mosquitoes collected at a fishpond in south Moravia, Czech Republic, with a minimum infection rate of 0.04%. This alphavirus was never detected in Central Europe before.

## 1. Introduction

Mosquito-borne alphaviruses (genus *Alphavirus*; family *Togaviridae*) have often been reported to cause disease outbreaks worldwide [1]. In the past few decades, single or large epidemic events were caused by Chikungunya virus (CHIKV) in Europe, Asia, the Americas, and Pacific islands; Sindbis virus (SINV) in Northern Europe; Ross River (RRV) and Barmah Forest viruses (BFV) in Australia; Mayaro virus (MAYV) in South America; or O’nyong’nyong virus (ONNV) in Africa [2]. Pathogenic alphaviruses generally cause fever, headache, rash, fatigue, muscle pain, arthralgia, and/or arthritis, less frequent symptoms include nausea, dizziness, enlarged lymph nodes, diarrhea, and photophobia. Some patients, mostly in the case of SINV fever, experienced prolonged joint symptoms (mainly arthritis) over months or even years after infection. Infections are often self-limited or subclinical, which might contribute to their underdiagnosis and, thus, limited availability of routine clinical testing [3,4]. As a consequence, the eco-epidemiology of mosquito-borne alphaviruses and actual burden of diseases in human population is still not completely understood [1,2,3].

The mosquito *Culex modestus* Ficalbi, 1889 (Diptera: *Culicidae*) is a Eurasian mosquito species that is distributed from England to southern Siberia and common in southern and central countries of Europe. Typical larval habitats of *Cx. modestus* constitute ponds and swamps with rich vegetation, marshes, flooded wetlands, ditches, and rice fields [5]. In the Czech Republic, the species has been frequently reported from South Moravia and South Bohemia, where both larval and adult stages have been observed from early June to late September, typically in reed beds surrounding fish ponds [6,7], and often syntopically with *Anopheles hyrcanus* Pallas, 1771, *An. maculipennis* Meigen, 1818, *Cx. pipiens* Linnaeus, 1758, and *Uranotaenia unguiculata* Edwards, 1913 [7]. 

Several surveillance studies have been aimed at the detection of pathogenic flaviviruses and bunyaviruses in a particular mosquito vector [8,9]. However, despite the fact that several members of this group, namely SINV or Western and Eastern equine encephalitis viruses, circulate within a mosquito–bird cycle [3], similarly to West Nile virus (WNV) and Usutu virus (USUV), both of which are established in *Cx. modestus* mosquitoes in Central Europe [8,10], only few studies have focused directly on alphaviruses. Therefore, we aimed at the molecular screening of *Cx. modestus* mosquito females for pathogenic alphaviruses, which might (in addition to WNV and USUV) contribute to public health burden of mosquito-borne viral diseases in Central Europe. 

## 2. Materials and Methods

### Mosquito Trapping, Their Identification, and Processing

In this study, *Cx. modestus* mosquitoes were collected in reedbeds along shore of the fishpond “Mlýnský” (48°47′ N, 16°49′ E; 86 ha, 162 m a.s.l.) near Lednice in South Moravia, Czechland. They were captured using CDC miniature light‒CO_2_ (dry ice)-baited traps during two seasons, 2010 (July 15 to October 14) and 2014 (July 30 to August 28). The trapped insects were then transported to the laboratory in cooled flasks and stored at −65 °C until examination. Mosquito species and sex were determined on a cooled plate under a stereomicroscope [5]. Monospecific pools of 50 females of *Cx. modestus* were prepared. They were homogenized in 1.5–2.0 mL of cooled phosphate-buffered saline, pH 7.4, supplemented with 0.4% bovine serum albumin fraction V (Sigma, Saint Louis, MO, USA), penicillin (500 i.u./mL), streptomycin (100 μg/mL), and gentamicin (100 μg/mL). The homogenates were centrifuged at 1500× *g* for 20 min (at 0 °C), and supernatants were used for RNA extraction and viral isolation attempts. The RNA was extracted from 150 μL of mosquito homogenates by using the QIAamp viral RNA Mini Kit (Qiagen, Hilden, Germany) according to the manufacturer’s instructions. All suspensions were tested by broad-range reverse transcriptase-polymerase chain reaction (RT-PCR) for alphaviruses using generic primers assigned as “VIR966” [11]. Alphavirus-positive pools were then tested with primers targeting three different fragments, specifically positions 8122 to 8885, 9415 to 10,044, and 9978 to 10,642 of Sindbis virus polyprotein gene, encompassing E2 and E1 regions [12]. A continuous RT-PCR system employing the QIAGEN OneStep RT-PCR Kit (Qiagen, Hilden, Germany) was applied on RNA extracts. Specific PCR products were further processed by the BigDye Terminator v3.1 Cycle Sequencing Kit (Applied Biosystems, Foster City, CA, USA) and characterized by sequence analysis (Sanger method) on an ABI PRISM 310 Genetic Analyzer (Applied Biosystems, Foster City, CA, USA). Obtained sequences were compared by a basic alignment search tool (BLAST) and further aligned with a partial nucleotide sequences of particular protein coding region and/or complete genome sequences of other Sindbis virus strains deposited in GenBank database. The evolutionary history was inferred by the maximum-likelihood method (ML) using Mega 7.0 [13]. The best-fit model of molecular evolution was determined for the analysis dataset using the Akaike Criterion (AIC) in Modeltest ver. 2.1.4 [14]. For ML analysis, we conducted heuristic searches under a GTR + G (general time-reversible model). Initial tree(s) were obtained automatically by applying neighbor-join and BioNJ algorithms to a matrix of pairwise distances estimated using the maximum-composite-likelihood (MCL) approach, and then selecting the topology with superior log likelihood value. A discrete gamma distribution was used to model evolutionary rate differences among sites (G = 0.9441). The tree with the highest log likelihood −10,918.66) is shown. The robustness of inferred trees was assessed by bootstrapping (1000 replicates).

Pools positive for *Alphavirus* RNA in PCR were tested on suckling mice by intracerebral inoculation (0.02 mL). The experiments with laboratory mice were conducted in accordance with the Czech Animal Protection Act no. 246/1992, and the protocols were approved by the Institutional and Central Care and Use Committees at the Academy of Sciences of the Czech Republic in Prague. The facility is accredited by the Czech National Committee on Care and Use of Laboratory Animals (70084/2016-MZE-17214).

## 3. Results and Discussion

**Figure 1:**
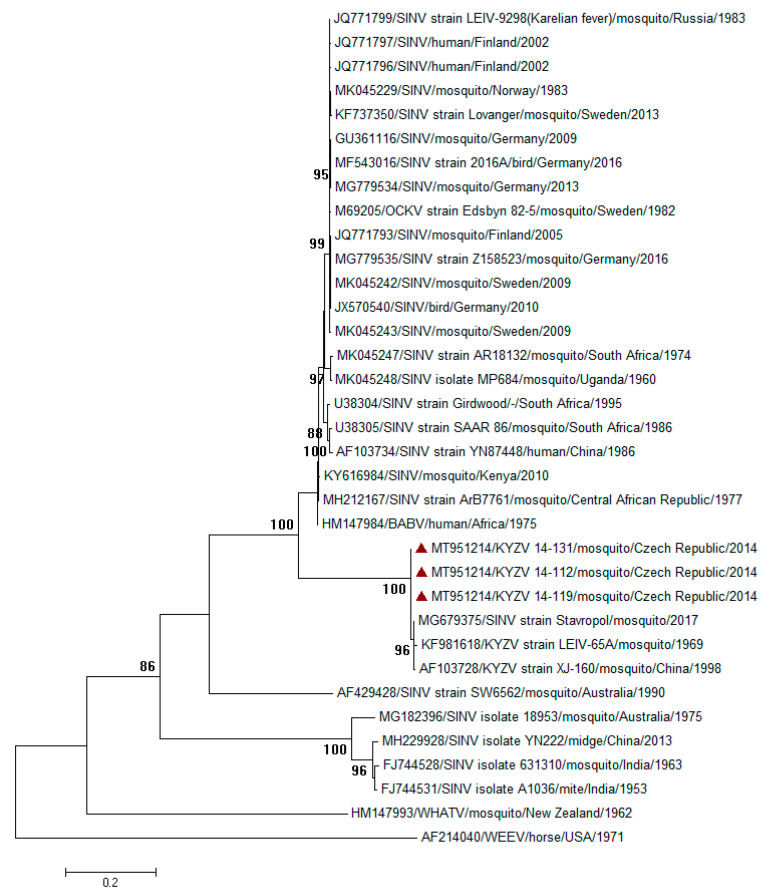
Phylogram demonstrating relationship of Kyzylagach virus (KYZV) detected in mosquitoes in the Czech Republic, based on a 1992-bp-long partial nucleotide sequence of the virus polyprotein gene from Czech sequences obtained in this work and other Sindbis virus strains circulating worldwide. Each record consists of particular accession number, source (human/mosquito/bird), place, and year of detection/isolation. Czech samples are highlighted by red triangles. Phylogenetic analyses were conducted using the maximum-likelihood (ML) algorithm using the general time-reversible model (MEGA 7.0). The robustness of trees was tested by bootstrap resampling of 1000 replicates, and its values are listed near the nodes (only values ≥85 are shown). The horizontal bar shows genetic distance. (Legend: BABV—Babanki virus; KYZV—Kyzylagach virus; OCKV—Ockelbo virus; SINV—Sindbis virus; WHATV—Whataroa virus; WEEV—Western equine encephalitis virus).

Females of *Cx. modestus* mosquitoes collected in 2010 (4487 individuals in 90 pools) were all negative, while four pools collected in 2014 (6297 individuals in 131 pools) were positive for Kyzylagach virus (KYZV). The minimum infection rate is therefore 0.06%. However, no RNA-positive pool killed suckling mice. According to phylogenetic analysis of a 1992-bp-long fragment of the polyprotein gene (encompassing E2 and E1 regions) (Figure 1), we can conclude that our strains share sequence similarity (98.2% nucleotide identity) with the strain KYZV LEIV-65A (GenBank accession no. KF981618), strain Stavropol (MG679375) as well as Chinese strain XJ-160 (AF103728). Moreover, all obtained sequences were identical. The Czech representative sequence was deposited in the GenBank database under accession no. MT951214.

A prototype strain of KYZV was first isolated from *Cx. modestus* mosquitoes collected in the Kyzyl-Agach game preserve (39°03′ N, 48°50′ E) on the shores of the Caspian Sea in southeastern Azerbaijan in a breeding colony of ardeid birds on 16 August 1969 [15]. The virus has been named as Sindbis-like virus (subtype or variant of Sindbis virus, SINV: [16,17]). A virtually identical strain (XJ-160) of KYZV was isolated from *Anopheles* sp. mosquitoes captured in a rice field at the Yili River in Xinjiang, China in 1990 [18], with only 0.01% difference in nucleotides and amino acids between strains LEIV-65A and XJ-160 [19]. KYZV was later assigned to Sindbis virus genotype 4 using genome sequencing data [19,20,21]. Based on phylogenetic analysis, an additional five SINV genotypes were identified: Sindbis virus genotype 1 (SINV-1) is distributed in Europe, Africa, and the Middle East; Sindbis virus genotype 2 (SINV-2) and Sindbis virus genotype 6 (SINV-6) in Australia; Sindbis virus genotype 3 (SINV-3) in Southeast Asia; and Sindbis virus genotype 5 (SINV-5) (also referred to as Whataroa virus) in New Zealand. Human infections including documented epidemics are attributed in most cases to the SINV-1 genotype [20,22]. 

According to literature [15,23] both SINV and KYZV kill newborn mice when inoculated intracerebrally, but newborn mice are not susceptible to intraperitoneal inoculation of KYZV, whereas they are killed after the same route of inoculation with SINV. There is also only a one-way antigenic cross-reaction between these viruses: KYZV antibodies do not neutralize SINV, while SINV antibodies neutralize both viruses in a plaque-reduction neutralization test [16]. The same one-way reaction was observed in a complement fixation test [23].

**Figure 2:**
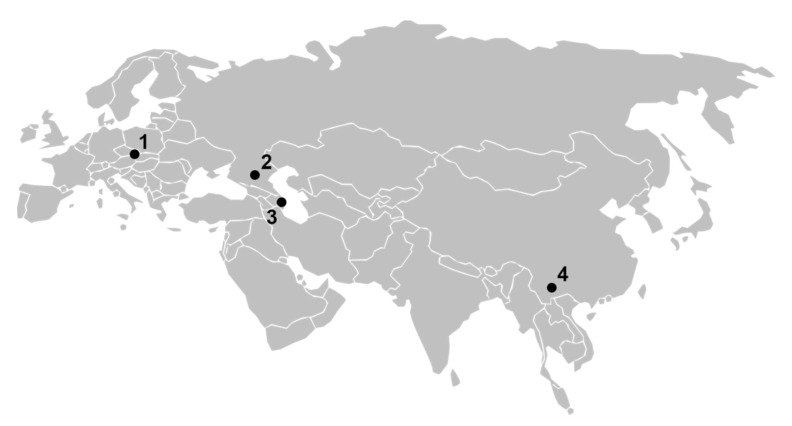
Current geographical distribution of Kyzylagach virus: 1—South Moravia (Czech Republic); 2—Stavropol region (southern Russia); 3—Kyzylagach preserve (Azerbaijan); 4—Yili river, Xinjiang (China).

The current distribution of KYZV includes four geographically distant areas—Czech Republic, southern Russia, Azerbaijan, and China (Figure 2). Calisher et al. [16] wrote “Viruses of the WEE complex (including Sindbis virus) with lesser antigenic differences may develop in discrete ecologic conditions,” which may be also valid for KYZV. In accordance with a previous study [15], the vector of KYZV is most likely *Cx. modestus* occurring predominantly in wetland habitats with reed beds and abundant water birds across Europe and Asia. The Kyzyl-Agach game preserve, the place where the KYZV prototype strain was originally isolated, is the most important migration stopover site for migrating waterfowl in the former Soviet Union (geographically and politically belonging to Azerbaijan). During wintertime, coots, ducks, geese, swans, and flamingo are found in the ice-free freshwater coves and shallows of the sea. During the summer, large colonies of glossy ibises, squacco herons, egrets, and purple herons occur. Similarly, a local (Czech) study site is characterized by the typical reed bed ecosystem (*Phragmites communis* alliance) situated at the littoral zone of the fishponds. More than 30 species of birds have been recorded breeding in the reed bed in southern Moravia, and an additional 54 wild wetland and terrestrial bird species visit this ecosystem during seasonal movements [24]. This might contribute to transport of mosquito-borne KYZV over long geographical distances.

From an epidemiological point of view, *Cx. modestus* is a very efficient vector of West Nile virus (WNV) [8,25,26] and is implicated in the circulation of two other local arboviruses, Ťahyňa and Lednice viruses [27,28]. The females of *Cx. modestus* feed preferably on birds (e.g., Anseriformes), but sometimes feed on mammals (e.g., horses and rabbits) and may therefore act as bridge vectors for certain arboviruses [29,30,31]. They are also reported to commonly bite humans outdoors, close to their larval habitat [5]. *Cx. modestus* might be a frequent human-biting species in certain areas of the United Kingdom [32].

From a medical point of view, KYZV antibodies were previously found in a Chinese human population. A seroprevalence study carried out with the strain XJ-160 involving 521 subjects from 10 different provinces in China showed that 19% of the subjects had positive antibody titers against the XJ-160 strain [18]. Partial cross-reaction with very closely related SINV-1 cannot be excluded. In fact, SINV-4 has never been reported associated to human disease.

In conclusion, future surveillance efforts in Central Europe for mosquito-borne viruses of medical importance should take into account variant Sindbis viruses, in addition to SINV, WNV, USUV, Ťahyňa, and Batai arboviruses. Studies on alphaviruses have been neglected or they have received less attention in Central Europe.
